# Effect of psychoeducation and telepsychiatric follow up given to the caregiver of the schizophrenic patient on family burden, depression and expression of emotion

**DOI:** 10.12669/pjms.295.2613

**Published:** 2013

**Authors:** Birgul Ozkan, Emine Erdem, Saliha Demirel Ozsoy, Gokmen Zararsiz

**Affiliations:** 1 Birgul Ozkan, PhD, Lecturer, Department of Nursing, Faculty of Health Sciences, Erciyes University, Kayseri, Turkey.; 2 Emine Erdem, PhD, Assistant Professor, Department of Nursing, Faculty of Health Sciences, Erciyes University, Kayseri, Turkey.; 3 Dr. Saliha Demirel Ozsoy, Associate Professor, Department of Psychiatry, Faculty of Medicine, Erciyes University, Kayseri, Turkey.; 4 Gokmen Zararsiz, MSc, Research Assistant, Department of Biostatistics, Faculty of Medicine, Erciyes University, Kayseri, Turkey.

**Keywords:** Family burden, Psychoeducation, Patients with schizophrenia, Telepsychiatry

## Abstract

***Objective: ***This randomized-controlled experimental study was conducted to determine the effects of psychoeducation in the inpatient clinic and regular telepsychiatric follow-up (via telephone) after discharge on emotional expression, depression and family burden of primary caregivers of the schizophrenic patients.

***Methods:*** This study was performed on 62 caregivers of the schizophrenic patients, assigned to experiment (n=32) and control (n=30) groups, from 1^st ^July 2010 to 31^st^ May 2011. The Scales for Expressed Emotion, the Beck Depression and the Zarit Family Burden were applied for the caregivers in experiment and control groups before education, after education and after 6-months telephone follow-up.

***Results: ***The mean scores of the caregivers in experiment group on family burden, emotional expression and depression were decreased after education and after telephone follow-up and difference was significant (p<0.001).

***Conclusions*** *: *Psychoeducation and telepsychiatric follow-up via telephone induced decrease in family burden, emotional expression and depressive symptoms for their caregivers and was a support for the family in the patient care.

## INTRODUCTION

Family interventions are accepted as an important part of modern treatment methods besides drugs in schizophrenia treatment and care.^1^^,^^2^ Most family intervention programs include cognitive therapy techniques and psychoeducation. Psychoeducation promotes the development of the coping abilities of the patients and caregivers by providing information about treatment and care of the mental disorders.^3^^,^^4^ McWills et al found that patients whose caregivers learned more from the six-week psycho-education course had a significantly longer time to relapse and shorter length of stay during their first relapse.^5^

Several studies have demonstrated that patients in families having high levels of expressed emotion (EE), depression and family burden are more likely to experience a clinical relapse than patients in families with low levels of EE, depression and family burden. Caring for people with psychosis has been associated with subjective burden and loss, depression, distress, reduced quality of life, lower social support.^6^^,^^7^

Education given to family members, particularly primary caregivers, about disease, symptoms and drug compliance and follow-ups at home are important for the home care of schizophrenic patients.^6^ Several clinical trials have demostrated that brief psycho-education programmes can improve symptomatic recovery, reduce relapse and enhance short-term outcomes for psychosis and schizophrenia.^5^^-^^7^

Follow-up by phone is considered to be an innovative model that may facilitate home care services for patients with mental disorders, and difficulties in patient care may be reduced by this method.^8^^,^^9^ There are studies which have indicated that telepsychiatric services can be used in clinical care, education and researches.^10^^,^^11^ Telepsychiatric services may be provided through many ways; such as video conference, e-mail, website and phone.^12^^,^^13^

In Turkey’s health services, treatment and care of schizophrenic patients are provided only during hospitalization and home care cannot be provided after discharge. There are few studies related to EE, depression and family burden in caregivers of schzophrenic patient in Turkey. Therefore, these studies are needed.

This study was designed to determine the effect of psychoeducation given to the caregivers of schizophrenic patients in the clinic and telepsychiatric follow-up provided by regular phone, on their expression of emotions, depression and family burden levels.

## METHODS

This study was designed as a randomized controlled experimental study (experimental-control groups with pre-test and post-test) between 01 July 2010 and 31 May 2011 and was approved by the Ethics Committee. The hospitalized patients which fulfileld the inclusion criteria were given the odd numbers for the experimental and dual numbers for the control group.

The research was conducted with primary caregivers of schizophrenic patients who were hospitalized and followed-up in the psychiatric clinic in Kayseri, Turkey. The sample consisted of 62 caregivers (experimental group=32, control group = 30). Following the data collection, PASS 11 (Power Analysis & Sample Size Software) was used to determine the number of samples in each group, α=0.05, power analisis= 100% in the all scales found. The research was conducted randomly with a sample set of criteria which was age, gender, degree of relationship to patient, educational, marital ,working and economic status of the caregivers. After identifying the experimental and control group all the scales were applied to both groups.

The Level of Expressed Emotion (LEE) Scale, developed by Cole & Kazarian^7^ and adapted to Turkish by Berksun^14^ is applied to the caregivers. High scores indicate high level of negative expression of emotions. The Beck Depression Inventory (BDI), comprised of 21 questions which was developed by Beck et al^15^ and adapted to Turkish by Hisli.^16^ High scores indicate a high level of depression

The Zarit Family Burden Assessment Scale was developed by Martin^17^ and adapted to Turkish by Ozlu et al.^18^ High scores indicate high family burden. Telepsychiatric follow-up of caregivers was carried out with the Follow-Up by Phone Form for Caregivers of Schizophrenic Patients.

All scales used in this study were filled out by the researcher through face to face interviews with the caregivers in the experimental group during the hospitalization of their patients (before education), on discharge (after education), and after the 6-month follow-up by phone. The caregivers in the experimental group were given psychoeducation through face to face interviews in 8 sessions during the hospitalization of their patients. The education sessions included topics about the disease and disease management which took an average of 35-50 minutes. No interventions were made for the caregivers in the control group during survey. However, psychoeducation were made for the caregivers in the control group after study. After discharge, the caregivers in the experimental group were followed-up with telephone calls which took an average of 15 minutes on standard days and hours throughout six months. Followed-up with telephone were not made for the caregivers in the control group. The qui-square test, the student’s t, the repeated measures two way analysis of variance Bonferroni and LSD tests were used in statistical analysis. Chi-square analysis was performed for categorical comparisons. A two-way repeated measures analysis of variance was applied followed by Bonferroni and LSD tests to examine the effect of group, time and groupxtime interaction to all mean scores. Analysis were conducted using IBM SPSS Statistics 20.0 (Chicago, ILL, USA) software. A p value less than 0.05 was considered as statistically significant.

## RESULTS

Age, gender, relationship to patient , educational, marital, working and economic status of the caregivers in the experimental and the control groups were similar and there was no statistically significant difference between groups (p>0.05) (Table-I.).

 The mean scores of expressed emotion scale of the caregivers in the experimental and the control groups were analyzed. A significant difference was seen between repeated measurements (p<0.001). It was determined that the difference was between before and after the education (on discharge), and before the education and after the telephone follow-up phone call in the experimental group; the difference between all measurements in control group was found (p<0.05) (Table-II).

 The mean scores of depression of the caregivers in the experimental and the control groups were analyzed. A significant difference was detected between the the repeated measurements (p<0.001). The difference was determined between before education and after education, before education and after telephone follow-up phone calls, and after the education, after telephone follow-up in the experimental group, and the before education and after education in the control group (p<0.05) (Table-III).

 A significant difference was detected between the repeated measurements (p<0.001). The difference was determined between the before education and after education, before education and after telephone follow-up, and after education and after telephone follow-up phone in the experimental group (p<0.05) (Table-IV).

**Table-I:** Characteristics of caregivers in experimental and control groups

*Introductory properties*		*Experimental group* * (n=32)*					*Control * *group* * (n=30)*					*Tests*	*p*	
		S			%		S			%				
*Gender*														
Female		14		43.7			19		63.3			X^2^=1.663	0.197	
Male		18		56.3			11		36.7					
*Years*														
26-35		4		12.5			4		13.3			X^2^=1.258	0.214	
36-45		10		31.2			9		30.1					
46-55		12		37.5			10		33.3					
56 +		6		18.8			7		23.3					
*Marital Status*														
Married		25		78.1			25		83.3			X^2^=3.272	0.352	
Widowed		5		15.6			1		3.3					
Divorce		1		3.1			2		6.7					
Single		1		3.1			2		6.7					
*Working Status*														
Employed		13		40.6			7		23.3			X^2^=1.401	0.237	
Unemployed		19		59.4			23		76.7					
*Educational Status*														
Primary School		13		40.6			12		40.0			X^2^=0.720	0.869	
Secondery School		9		28.1			9		30.0					
High School		6		18.8			7		23.3					
Universty		4		12.5			2		6.7					
*Economic Status *													
Affluent	2		6.2			-		-			X^2^=2.478		0.298
Middle class	26		81.3			24		80.0					
Poor	4		12.5			6		20.0					
*Degree of relationship to patient*													
Parents	8		25.0			7		23.3			X^2^=4.473		0.215
Brotherhood/Sisterhood	14		43.8			10		33.4					
Child	4		12.5			1		3.3					
Husband/Wife	6		18.7			12		40.0					
*Total*	32		100			30		100					

**Table-II:** The mean scores of expressed emotion scale of the caregivers in the experimental and the control groups before, after the education and after telephone follow-up

*Groups*	*Time*	*Mean * *Scores of* *Expressed Emotion* *Scale *					
		*Overconcern/Over protectiveness * *x ± SS*		*Criticim/Hostility* *x ± SS*		*General Point* *x ± SS*	
Experimental	Before education After education After follow-up	9.21±1.43^ a^ 6.93±2.15^ b^ 6.28±1.83^ b^		23.96±5.33^ a^ 15.06±4.03^ b^ 14.12±4.01^ b^		33.18±6.16^ a^ 22.00±5.34^ b^ 20.40±5.56^ b^	
Control	Before education After education After follow-up	6.23±1.92^ a^ 7.53±1.85^ b^ 9.20±2.49^ c^		20.16±3.09^ a^ 20.96±2.72^ b^ 23.76±1.85^ c^		26.40±4.68^ a^ 28.50±4.16^ b^ 32.96±3.24^ c^	
		KO	F	KO	F	KO	F
	Time	10.237	5.400	301.815	26.564	299.002	13.178
	Time+Group	269.878	59.659	1399.234	123.152	2898.131	127.726
	Group	1.445	0.276	711.823	31.583	777.417	18.821
Test	P* P+ P#	<0.001 <0.001 <0.001		<0.001 <0.001 <0.001		<0.001 <0.001 <0.001	

P* = inter-group, P+ = between measurements/times, P# = group and time interaction

The results of in-group multi-comparisons of the experimental and control groups were displayed with alphabethic superscripts; the same letters indicating an insignificant statistical difference between the measurement times and different letters indicating a statistically significant difference.

**Table-III:** The mean scores of the Beck Depression Inventory of the caregivers in the experimental and the control groups before, after education and after telephone follow-up

*Groups*	*Time*	*Beck Depresyon Puani* *x ± SS*	
Experimental	Before education After education After follow-up	26.09±8.56^ a^ 17.40±7.12^ b^ 12.68±7.19^ c^	
Control	Before education After education After follow-up	40.56±7.78^ a^ 43.30±7.56^ b^ 43.43±9.20^ b^	
		KO	F
	Time	859.996	27.733
	Time+Group	2050.125	66.122
	Group	26100.581	173.868
Test	P* P+ P#	<0.001 <0.001 <0.001	

P* = inter-group, P+ = between measurements/times, P# = group and time interaction

The results of in-group multi-comparisons of the experimental and control groups were displayed with alphabethic superscripts; the same letters indicating an insignificant statistical difference between the measurement times and different letters indicating a statistically significant difference.

**Table-IV:** The meanscores of Zarit Family Burden Scale of the caregivers in the experimental and control groups before, after education and after telephone follow-up

*Groups*	*Time*	*Family Burden General Point* *x ± SS*	
Experimental	Before education After education After follow-up	75.65±14.35^ a^ 54.96±13.54^ b^ 46.18±14.33^ c^	
Control	Before education After education After follow-up	96.46±11.95^ a^ 93.36±7.14^ a^ 95.46±5.61^ a^	
		KO	F
	Time	7187.185	76.203
	Time+Group	6274.604	66.527
	Group	60746.001	229.629
Test	P* P+ P#	<0.001 <0.001 <0.001	

P* = inter-group, P+ = between measurements/times, P# = group and time interaction

The results of in-group multi-comparisons of the experimental and control groups were displayed with alphabethic superscripts; the same letters indicating an insignificant statistical difference between the measurement times and different letters indicating a statistically significant difference.

## DISCUSSION

The fact that the patients have to live with their families brings along many problems. However, the effect of the disease on the family is ignored.^2^^-^^5^ In the study of Kavanagha,^19^ which was carried out with the families of schizophrenic patients, he found that relapses increased in the patients of families whose expressed emotion level was high. Insufficient or incorrect knowledge of the family about the disease, its treatment and family attitudes affect their behaviours towards the patient and the symptoms of the disease.^20^

 In the current study, the mean scores of expressed emotion of the caregivers in the exprimental group which were higher than that of the control group before the psychoeduction were found to have significantly decreased after the education and telephone follow-up phone calls. Furthermore, disease management, which began with psychoeducation and was supported by telephone follow-up, being informed about the correct use of the drugs and awareness may have positively affected the expressed emotions of the caregivers.

 In the study of Cohan et al^2^, psychosocial education was found to reduce the level of expressed emotion of the caregivers of schizophrenic patients. The results of other studies are similar to the results of this study.^7^^,^^8^ In the study of Holmes et al^21^ carried out with 17 schizophrenic patients and their families, they did a one-year follow-up after the individual psychoeducation provided by the psychiatry nurse considering the patients’ characteristics, and found that awareness about the disease and social functioning increased, and that the high level of expressed emotion of the family decreased.

 Relatives of schizophrenic patients feel decreased self esteem respect, hopelessness, helplessness and weakness. As a result of these negative feelings, their likelihood of depression may increase in the relatives, particularly the primary caregivers of the patient.^5^^,^^6^^,^^10^^,^^13^

 In the current study, it was found that the depression scores of the caregivers which were high before the psychoeducation significantly decreased after the education and telephone follow-up in the experimental group; the depression scores of caregivers in control group increased in the future stages (Table-II). Being informed and aware of disease management, regular use of the drugs, counseling through telephone follow-up may have enabled the caregivers to manage the treatment and care of the patients and hence, a decrease in the mean scores of depression.

 In care burden studies carried out with the relatives of schizophrenic patients, more contact between caregivers and the patients, living with their families were reported to increase the level of care burden.^12^^,^^22^^-^^24^ In the study of Magliano et al.^25^, which was comparing the care burden of the families of schizophrenic patients (n=709) and patients with chronic physical diseses (n=709), they determined that the subjective burden of the caregivers of schizophrenic patients was increased, and the extent of social support and support in case of emergency were decreased.

 With telephone follow-up; it was determined that social isolation and the stress level of the patient and the family were decreased. It was also reported that the care and education requirements of the patient and the family are more effective, economical and easy.^2^^,^^11^^,^^13^^,^^20^^-^^22^ Similarly, in our study, the family burden scores of the caregivers in the experimental group which were high before the psychoeducation were found to decrease after the psychoeducation and this decrease continued after telephone follow-up; however, the mean scores of the control group did not change significantly.

 It was reported that family interventions (psychoeducation and follow-up) provided for caregivers decreased the levels of depression, expressed emotion and family burden of caregivers.^2^^-^^4^^,^^6^^,^^10^^-^^13^^,^^23^^,^^24^ These findings are consistent with the results of our study.

***Limitations and future prospects: ***This study has some limitations and several strengths. The sample size was large enough to allow adequate statistical power to clearly determine the intervention’s effectiveness. The use of randomization allowed to obtain two groups of patients with very similar baseline characteristics.

## CONCLUSION

Psychoeducation and telepsychiatric follow-up via telephone decreased the levels of the family burden, emotional expression and depression in the caregivers and supported the family in patient care.
